# Characterizing Two Inter-specific Bin Maps for the Exploration of the QTLs/Genes that Confer Three Soybean Evolutionary Traits

**DOI:** 10.3389/fpls.2016.01248

**Published:** 2016-08-23

**Authors:** Wubin Wang, Meifeng Liu, Yufeng Wang, Xuliang Li, Shixuan Cheng, Liping Shu, Zheping Yu, Jiejie Kong, Tuanjie Zhao, Junyi Gai

**Affiliations:** ^1^Soybean Research Institute, Nanjing Agricultural UniversityNanjing, China; ^2^National Center for Soybean Improvement, Ministry of AgricultureNanjing, China; ^3^Key Laboratory of Biology and Genetic Improvement of Soybean (General), Ministry of AgricultureNanjing, China; ^4^State Key Laboratory for Crop Genetics and Germplasm Enhancement, Nanjing Agricultural UniversityNanjing, China; ^5^BGI-Shenzhen, Beishan Industrial ZoneShenzhen, China; ^6^Jiangsu Collaborative Innovation Center for Modern Crop Production, Nanjing Agricultural UniversityNanjing, China

**Keywords:** wild soybean, linkage map, days to flowering, seed coat color, seed bloom, restriction-site-associated DNA sequencing (RAD-Seq)

## Abstract

Annual wild soybean (*Glycine soja* Sieb. and Zucc.), the wild progenitor of the cultivated soybean [*Glycine max* (L.) Merr.], is valuable for improving the later. The construction of a linkage map is crucial for studying the genetic differentiation between these species, but marker density is the main factor limiting the accuracy of such a map. Recent advances in next-generation sequencing technologies allow for the generation of high-density linkage maps. Here, two sets of inter-specific recombinant inbred line populations, named NJIRNP and NJIR4P, composed of 284 and 161 lines, respectively, were generated from the same wild male parent, PI 342618B, and genotyped by restriction-site-associated DNA sequencing. Two linkage maps containing 5,728 and 4,354 bins were constructed based on 89,680 and 80,995 single nucleotide polymorphisms, spanning a total genetic distance of 2204.6 and 2136.7 cM, with an average distance of 0.4 and 0.5 cM between neighboring bins in NJRINP and NJRI4P, respectively. With the two maps, seven well-studied loci, *B1* for seed bloom; *G* and *I* for seed coat color; *E2*, *E3*, *qDTF16.1* and two linked *FLOWERING LOCUS T* for days to flowering, were detected. In addition, two SB and two DTF loci were newly identified in wild soybean. Using two high-density maps, the mapping resolution was enhanced, e.g., *G* was narrowed to a region of 0.4 Mb on chromosome 1, encompassing 54 gene models, among which only *Glyma01g40590* was predicted to be involved in anthocyanin accumulation, and its interaction with *I* was verified in both populations. In addition, five genes, *Glyma16g03030*, orthologous to *Arabidopsis Phytochrome A* (*PHYA*); *Glyma13g28810*, *Glyma13g29920*, and *Glyma13g30710* predicted to encode the *APETALA 2* (*AP2*) domain; and *Glyma02g00300*, involved in response to red or far red light, might be candidate DTF genes. Our results demonstrate that RAD-seq is a cost-effective approach for constructing high-density and high-quality bin maps that can be used to map QTLs/genes into such small enough regions that their candidate genes can be predicted.

## Introduction

Soybean [*Glycine max* (L.) Merr.], is one of the most economically important leguminous seed crops. Many important traits in soybeans were complex quantitative traits controlled by multiple QTLs (quantitative trait loci)/genes and environments. To improve the effectiveness of marker-assisted breeding in soybean, the mapping resolution needs to be enhanced. High-density genetic maps, one of the most valuable genomic resources, can largely reveal the genome composition and meet the above requirement. Thus, the construction of a high-density linkage map for soybean is necessary for its future study and production.

In the past two decades, there have been a number of reports on the construction of soybean genetic maps. As a permanent population, recombinant inbred lines (RILs) have been mainly used for this research in soybean because they can be evaluated over space and time and used indefinitely, facilitating the exchange, accumulation, and sharing of genetic information (Mansur et al., 1996). Using an RIL population from a cross between two soybean cultivars, an initial genetic map defining 1550 cM of the soybean genome comprising 31 linkage groups consisting of 132 RFLPs, isozymes, and morphological and biochemical markers was reported in Lark et al. (1993). Since this initial linkage map, researchers have developed more than 40 linkage maps. Our research group also made great advancements in linkage map construction and developed more than 10 maps, whose typical representative was composed of 452 markers in 21 linkage groups and covered 3,595.9 cM of the soybean genome (Zhang et al., 2004). The above genetic maps have provided tools for understanding the genetic bases of soybean quantitative traits but have mainly focused on cultivated soybeans, while only few studies have investigated inter-specific crosses, which facilitate the detection and use of favorable exotic genes to broaden the cultivar’s genetic bases.

Meanwhile, the marker densities of linkage maps were low, except for those of GmComposite2003 (Song et al., 2004) and GmConsensus40 (Hyten et al., 2010), which integrated five and three different populations, respectively. Recent advances in next-generation sequencing technologies have provided effective platforms for the detection of high-quality single nucleotide polymorphism (SNP) markers for the genotyping of mapping populations (Varshney et al., 2009), and genotyping-by-sequencing (GBS) has been successfully used for genetic studies in a variety of species (Wu et al., 2010; Chen et al., 2014; Li et al., 2015). However, the limitations to GBS include a relatively large proportion of missing data, and a small but rarely corrected percentage of SNP genotyping sequencing errors always occur during GBS. One option for imputing missing SNP data is the sliding-window approach, where adjacent SNPs with the same genotype in an interval are combined into bins that demarcate recombination locations across the whole population (Huang et al., 2009). The bin-map method is more powerful for detecting QTL than traditional methods and has been employed for the fine mapping of yield-associated loci in rice (Yu et al., 2011), maize (Chen et al., 2014), and others. In soybean, Xu et al. (2013) constructed the first bin maps using 246 RILs derived from a *G. max* cross between Magellan and PI 438489B and fine mapped a root-knot nematode resistance QTL into a bin of 29.7 kb. However, the advantages of high-density linkage mapping have not yet been demonstrated in the soybean inter-specific cross.

Annual wild soybean (*Glycine soja* Sieb. and Zucc.) is acknowledged as the wild progenitor of cultivated soybean (Broich and Palmer, 1980). During evolution, the seed bloom (SB) in the wild soybean disappeared, and the seed coat color (SCC) became yellow in the released cultivars. During adaptation to their environments, soybeans with variable days to flowering (DTF) were selected. So far, the genetic bases of these three evolutionary traits, SB, SCC and DTF, have been well-studied in cultivated soybeans. For SB, three genes, *B1*, *B2* and *B3*, have been reported (Palmer et al., 2004), while only *B1* has been mapped to a large region of chromosome (Chr.) 13 (Chen and Shoemaker, 1998). At least five genetic loci (*I*, *T*, *W1*, *R*, and *O*) are known to control SCC (Palmer et al., 2004), but they represent large intervals, except for *I*, which was located in a 103-kb gene-rich region harboring a cluster of chalcone synthase genes on Chr. 08 (Clough et al., 2004). For DTF, remarkable progress has been made in identifying genes, including *E1*–*E8* (Bernard, 1971; Buzzell, 1971; Buzzell and Voldeng, 1980; Mcblain and Bernard, 1987; Bonato and Vello, 1999; Cober and Voldeng, 2001; Cober et al., 2010), *J* (Ray et al., 1995), and QTLs (Mansur et al., 1996; Orf et al., 1999; Yamanaka et al., 2000; Tasma et al., 2001; Funatsuki et al., 2005; Reinprecht et al., 2006)^1^. However, the allele differentiations between wild and cultivated soybeans in the above evolutionary traits are largely unknown.

In the present paper, to improve the resolution of QTL mapping and knowledge of the evolutionary process from the wild soybean to cultivated soybean, two sets of ultra-high-density linkage bin maps derived from inter-specific crosses were constructed through restriction-site-associated DNA sequencing (RAD-seq). Thus, the genetic bases of three evolutionary traits, SB, SCC and DTF, were dissected. The results indicated that the availability of such a high-density linkage map would provide breeders and geneticists with a much-wanted tool to identify a smaller genomic region associated with the wild allele for a target trait and to analyze the allele differences between wild and cultivated soybeans to broaden the genetic bases of cultivated soybeans.

## Materials and Methods

### Plant Materials and Phenotyping

To analyze the genetic difference between wild and cultivated soybeans, two inter-specific populations, NJIRNP and NJIR4P, composed of 284 and 161 RILs, respectively, were developed by five cycles of single seed descent from F_2_ populations at the Jiangpu Experiment Station of Nanjing Agricultural University. NJIRNP was derived from a cross between Nannong86-4 (*G. max*) and PI 342618B (*G. soja*), while NJIR4P was derived from a cross between Nannong493-1 (*G. max*) and PI 342618B. Nannong86-4 and Nannong493-1, two cultivars in maturity group (MG) VI with days to maturity between 166 and 180 descripted by Gai et al. (2001), characterized by shiny SB, yellow SCC, later flowering, high yield, and high oil content were used as female parents, while PI 342618B, an annual wild soybean in MG I characterized by SB, black SCC, early flowering, high protein content, low 100-seed weight (1.1 g), and tolerance to flooding was used as a shared male parent.

NJRINP and NJIR4P, along with their parents, were grown in a complete randomized block design with three replications, one row per plot, and 100 cm between rows at Jiangpu Experiment Station in June 2010, 2011, 2012, and 2013 and at Anhui in 2012. Field management was performed under normal conditions. Three traits, SB, SCC and DTF, were investigated on a plot basis. For SB, the grading scales were B = Bloom (heavy coating of powdery substance adhering to the seed coat), S = Shiny (absence of bloom), and I = Intermediate (between bloom and shiny). For SCC, the grading scales were Y = Yellow, G = Green, Bl = Black, and Br = brown. DTF was scored as the days from sowing to the first flowering, which corresponded to the R1 developmental stage (Fehr et al., 1971).

### DNA Extraction and SNP Identification by Sequencing

The three parents were re-sequenced and the two RIL populations were RAD-sequenced at the Beijing Genomics Institution (Shenzhen, China). Genomic DNA from the fresh leaves of the two populations and parents was extracted using modified CTAB method (Murray and Thompson, 1980). Whole-genome re-sequencing was applied for the three parents. Sequencing libraries were constructed according to the manufacturer’s instructions (Illumina).

According to the protocol descripted by Chutimanitsakun et al. (2011) and Wu et al. (2014), the RAD-sequencing libraries were constructed to sequence the RIL population. Total of five main steps were included. First, 1 μg purified genomic DNA of each RIL was digested by 10 units of TaqI restriction enzyme, for 60 min at 37°C in a 50 μl reaction system, then the product was heat inactivated for 30 min at 65°C. Second, the 30 μl digestion products were ligated with 3.0 μl of 100 mM P1 adapter by 0.5 μl T4 DNA ligase (10 U/μl, NEB), along with 0.6 μl of 100 mM ATP, 6 μl 10× NEB Buffer, 5 μl nuclease free water and incubated at room temperature for 20 min. The P1 adapter was with the molecular identifier, sticky-end matching the TaqI cleavage site (TaqI-P1 top oligo: 5′-AATGATACGGCGACCACCGAGATCTACA CTTTCCCTACACGACGCTCTTCCGATCT ××××× TGCA-3′, TaqI-P1 bottom oligo: 5′-phos ××××× AGATCGGAAGAGCGTCGTGTAGGGAAGAGTGTAGATCTCGGTGGTCGCCGTATCATT-3′, ‘xxxxxx’ means molecular identifier). Third, every 24 RILs were pooled together and randomly sheared ultrasonically [Covaris S2 (Covaris, Inc.)], and QIAquick PCR Purification kit was applied for purifying sheared DNA fragments. The purified DNA was loaded on a 1.5% agarose gel with 100 bp DNA ladder and 400–600 bp DNA bands were cut from the gel and purified with MiniElute Gel Purification Kit (Qiagen). Fourth, the fragment end was repaired with Quick Blunting kit (NEB). A 3′-dA overhang was added using dA-tailing module (NEB). Then P2 adapter (top: 5′-phos-GATCGGAAGAGCGGTTCAGCAGGAATGCCGAGACCGATCAGAACAA-3′, bottom: 5′-CAAGCAGAAGACGGCATACGAGATCGGTCTCGGCATTCCTGCTGAACCGCTCTTCCGATCT-3′) was ligated to the sticky end with an overhanging A. Fifth, the collected fragments were enriched by PCR amplification (Forward primer: 5′-AATGATACGGCGACCACCGA-3′, reverse primer: 5′-CAAGCAGAAGACG GCATACGA-3′) and purified by QIAquick PCR purification kit. Finally, each sample was normalized to 10 nM, and was quantified on an Agilent 2100 Bioanalyzer and sequenced on HiSeq 2000 instruments following the manufacturer’s protocol.

Short reads were separated into each sample using a 6-bp barcode. All of the sequence reads were aligned against the reference soybean genome Wm82 (Schmutz et al., 2010) using SOAP2 (Li et al., 2009). SOAPsnp (version 1.01) was used for SNP calling in the three parents. For the RIL population, RealSFS (Yi et al., 2010) was used for SNP calling based on the Bayesian estimation of locus frequency. The software fastPHASE (Scheet and Stephens, 2006) was used for genotyping SNP imputations after heterozygous alleles were turned into missing alleles. The candidate SNPs were filtered with the following critical criteria: (1) SNPs existed between two parents; (2) sites with a probability >0.95; and (3) whole depth >40 and <2000. The final set of SNPs was used to construct bin markers.

According to the three parents’ sequences, small insertion and deletion (InDel) calling was performed according to a previously described method. Alignment was processed with SOAP2 allowing 5-bp gaps. A three-read-supported site was recognized as an InDel.

### RIL Genotyping and Bin Map Construction

High-density linkage maps of populations with high linkage disequilibrium contain many redundant markers that provide no new information but increase the computational requirements of mapping. Furthermore, a small percentage of genotypes are falsely called due to sequencing error. To address these issues, a modified version of the sliding-window approach developed by Huang et al. (2009) was applied. A sliding-window approach was used for RIL genotyping with a group of 15 consecutive SNPs and a step size of 2. The position of a SNP that switched one genotype to another consecutive genotype was recorded as the recombination breakpoint. A skeleton bin map was constructed according to the recombination breakpoint information. All of the breakpoints along the same chromosome were sorted by physical position. Consecutive 30-kb intervals that lacked a recombination event within the population were joined into bins, and the bins were used as markers. The linkage map was constructed using JoinMap 3.0 (van Ooijen and Voorrips, 2002) with Kosambi’s mapping function.

### Gene/QTL Mapping

For the two qualitative traits SB and SCC, the genes were mapped using the Chi-square test. The Bonferroni-corrected significance levels (0.05/bin number) were approximately 10^-6^ and 10^-5^ for NJRINP and NJRI4P, respectively. For DTF, the QTL analysis was performed on WinQTLCart 2.5 software (Wang et al., 2003) with the model of composite interval mapping. A 10-cM window at a walking speed of 1 cM was used in a stepwise forward regression procedure. The LOD threshold was calculated using 1000 permutations for an experimental-wise error rate of *P* = 0.05. The additive effects (Add) and phenotypic variance explained by QTL (*R*^2^) were estimated according to the bin at the highest peaks as determined by WinQTLCart 2.5. More than one QTL with overlapping one-lod-support intervals was treated as one QTL in the present paper.

## Results

### Establishment of the Genomic Bin-Marker System in the Two RIL Populations

#### SNP Identification in the Three Parents through Re-sequencing

With the whole-genome re-sequencing method, a total of 123.52, 134.42, and 110.36 M reads were generated for PI 342618B, Nannong86-4 and Nannong493-1, respectively. The short paired-end reads were mapped back to the Williams 82 reference genome (Schmutz et al., 2010) to determine the physical positions of each SNP. A total of 10.01, 11.27, and 9.13 Gb bases were identified, equivalent to a 9.89-, 10.69-, and 9.13-fold depth of PI 342618B, Nannong86-4 and Nannong493-1, respectively, with an average coverage of 86.48, 93.82, and 87.57% (Supplementary Table S1). Then, the genetic differences between the parents of the RIL populations were investigated. As shown in **Table 1**, total of 828,796 SNPs were identified between PI 342618B and Nannong86-4, while 736,076 SNPs were identified between PI 342618B and Nannong493-1. Only 251,612 SNPs were detected between Nannong86-4 and Nannong493-1. We also detected approximately 209,551 InDels between PI 342618B and Nannong86-4, while 162,452 were detected between PI 342618B and Nannong493-1. This shows that there was great genetic diversity between the cultivar and wild parents, with Nannong86-4 and PI 342618B having much more, as Nannong86-4 is an elite cultivar and Nannong493-1 is a landrace. These markers that were identified between parents of RILs are useful for QTL fine mapping.

**Table 1:** The genetic differences among the parents used to construct NJRINP and NJRI4P.

Chromosome	SNP		SV		Indel	
	P1 vs. P2	P3 vs. P2	P1 vs. P2	P3 vs. P2	P1 vs. P2	P3 vs. P2
Gm01	48,777	43,730	1,782	1,415	10,186	7,827
Gm02	36,767	39,310	1,706	1,522	10,579	8,770
Gm03	42,216	36,402	1,849	1,409	11,309	8,334
Gm04	43,410	41,930	1,636	1,393	9,234	7,560
Gm05	31,326	28,090	1,423	1,134	8,573	6,722
Gm06	44,095	36,059	2,164	1,621	13,207	9,619
Gm07	33,953	29,947	1,629	1,269	9,891	7,651
Gm08	35,737	32,660	1,450	1,148	10,592	8,822
Gm09	40,174	35,870	1,586	1,241	9,668	7,806
Gm10	43,607	38,446	1,627	1,307	10,138	8,296
Gm11	33,674	26,442	1,290	924	8,346	6,366
Gm12	34,918	32,479	1,381	1,187	8,043	6,666
Gm13	31,453	26,991	1,582	1,150	12,881	9,339
Gm14	46,955	43,124	1,842	1,599	9,256	7,740
Gm15	54,683	47,781	2,338	1,840	12,389	9,613
Gm16	35,507	29,239	1,653	1,219	10,492	7,450
Gm17	33,130	29,947	1,394	1,121	9,330	7,262
Gm18	66,143	56,828	2,828	2,280	15,128	10,824
Gm19	47,771	42,880	2,045	1,598	11,044	8,545
Gm20	44,500	37,921	1,715	1,366	9,265	7,240
**Total**	828,796	736,076	34,920	27,743	209,551	162,452

*P1, Nannong86-4; P2, PI 342618B; P3, Nannong493-1.*

#### SNP Identification of the RILs through RAD-seq

The method of RAD-seq was used to sequence each RIL. Restrict enzyme fragments ranging from 400 to 600 bp for 445 RILs were sequenced, generating a total of 1262.82 million paired-end reads (108.36 Gb), ~238.15 Mb for each line (Supplementary Figure S1). Paired-end reads were aligned to their respective parents. The complex genome composition with a high percent of repetitive DNA and nearly 75% of the genes existing in multiple copies might confuse read alignment, causing incorrect SNP calling; thus, only unique mapping reads were retained for subsequent analysis. The unique mapped sites accounted for approximately 4% of the whole genome, and the depth of each site was approximately fourfold in each individual on average. Although the unique mapping reads only covered approximately 4% of the whole genome, the SNP sets of the populations occupied up to 10% of the total SNPs between parents, that is 89,680/828,796 SNPs for NJIRNP and 80,995/736,076 SNPs for NJIR4P, approximately 10.8 and 12.2 kbp per SNP throughout the whole genome, respectively (**Table 2**; Supplementary Figures S2 and S3). The SNPs identified on different chromosomes ranged from 3,315 on Chr. 13 to 7,103 on Chr. 18, 2,534 on Chr. 16 and 6,104 on Chr. 19 in NJIRNP and NJIR4P (**Table 2**), respectively. This indicates that our sequencing method could capture high-density SNPs, as well as decrease the genome complexity.

**Table 2:** Distribution of SNPs on the 20 soybean chromosomes in the two RIL populations.

Chr.	NJRINP			NJRI4P		
	No.	Length (bp)	kbps/SNP	No.	Length (bp)	kbps/SNP
Chr. 01	5,333	55,915,594	10.5	5,447	55,915,594	10.3
Chr. 02	4,161	51,656,712	12.4	3,157	51,656,712	16.4
Chr. 03	4,732	47,781,075	10.1	3,577	47,781,075	13.4
Chr. 04	4,761	49,243,851	10.3	5,241	49,243,851	9.4
Chr. 05	3,527	41,936,503	11.9	3,741	41,936,503	11.2
Chr. 06	4,399	50,722,820	11.5	4,245	50,722,820	11.9
Chr. 07	3,721	44,683,156	12	3,100	44,683,156	14.4
Chr. 08	4,084	46,995,531	11.5	4,102	46,995,531	11.5
Chr. 09	3,712	46,843,749	12.6	3,538	46,843,749	13.2
Chr. 10	3,924	50,969,634	13	4,193	50,969,634	12.2
Chr. 11	3,703	38,408,601	10.4	2,748	39,172,789	14.3
Chr. 12	3,454	40,113,139	11.6	4,324	40,113,139	9.3
Chr. 13	3,315	44,408,970	13.4	2,675	44,408,970	16.6
Chr. 14	5,576	49,711,203	8.9	4,631	49,711,203	10.7
Chr. 15	5,982	50,939,159	8.5	5,174	50,939,159	9.8
Chr. 16	3,788	37,397,384	9.9	2,534	37,397,384	14.8
Chr. 17	3,853	41,906,773	10.9	3,600	41,906,773	11.6
Chr. 18	7,103	62,308,139	8.8	4,798	62,308,139	13
Chr. 19	5,549	50,589,440	9.1	6,104	50,589,440	8.3
Chr. 20	5,003	46,773,166	9.3	4,066	46,773,166	11.5
**Total**	89,680	949,304,599	10.8	80,995	950,068,787	12.2

*Chr., chromosome; No., number of SNPs identified in the NJRINP and NJRI4P populations derived from the crosses of Nannong86-4 × PI 342618B and Nannong493-1 × PI 342618B, respectively.*

#### Establishing a Bin-Marker System in the Two RIL Populations

**FIGURE 1:**
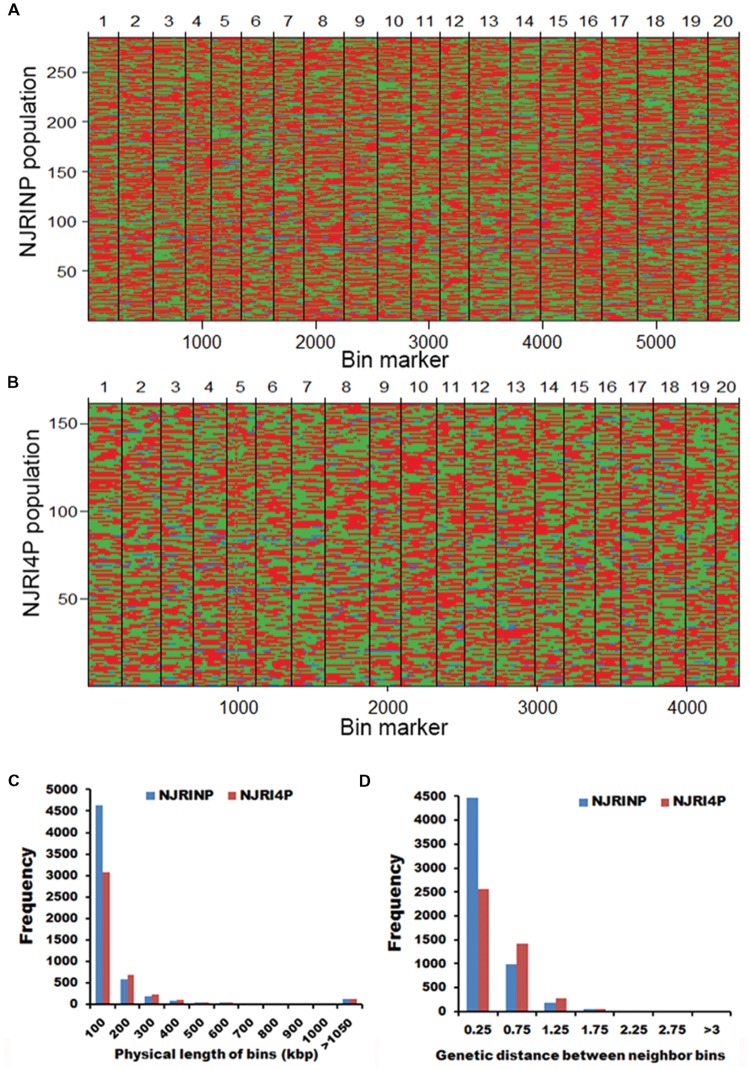
**Recombination bin maps of NJRINP and NJRI4P populations. (A)** Bin map of NJRINP derived from the cultivated soybean Nannong86-4 and wild soybean PI 342618B. The bin map consists of 5,728 bin markers inferred from 89,680 high-quality SNPs in 284 RILs. The physical position is based on the reference genome Williams 82. Chromosomes are separated by vertical lines. Red, green, and blue represent Nannong86-4, PI 342618B and heterozygote genotypes, respectively. **(B)** Bin map of NJRI4P derived from the cultivated soybean Nannong493-1 and wild soybean PI 342618B. The bin map consists of 4,354 bin markers inferred from 80,995 high-quality SNPs in 161 RILs. Red, green, and blue represent Nannong493-1, PI 342618B and heterozygote genotypes, respectively. **(C)** Distribution of the physical lengths of the bins in the NJRINP and NJRI4P populations. **(D)** Distribution of the genetic distances between neighboring bins in the NJRINP and NJRI4P populations.

It is difficult to construct a genetic map with more than 80,000 SNPs. A bin map is one of the most effective methods to decrease work and increase efficiency. As described in the methods, a sliding-window approach with a group of 15 consecutive SNPs was chosen to determine recombination breakpoints. According to the breakpoint information of each line, we identified 5,728 and 4,354 bin markers in NJRINP and NJRI4P (**Figures 1A,B**), respectively, which was more than that of the intra-specific RIL population (3,509 bins) identified using whole-genome re-sequencing technology (Xu et al., 2013). In NJRINP, the length of the bin markers ranged from 1.5 kb to 17.7 Mb, with a mean of 165.8 kb, and 80.9% of the bin markers were less than 150.0 kb in length, among which 116 bins that were larger than 1.05 Mb in size and two large bins that were more than 10.0 Mb were dispersed on Chr. 08 (bin2145) and Chr. 19 (bin5230; **Figure 1C**; Supplementary Table S2). In NJRI4P, the length of the bin markers ranged from 30.0 kb to 18.9 Mb, with a mean of 218.2 kb, and 70.8% of the bin markers were less than 150.0 kb in length, among which 125 bins that were larger than 1.05 Mb in size and three large bins that were greater than 10.0 Mb were dispersed on Chr. 08 (bin1798), Chr. 18 (bin3875), and Chr. 19 (bin4044; **Figure 1C**; Supplementary Table S2).

For NJRINP, 51.80% of all genotypes were inherited from the maternal parent Nannong86-4, 45.54% were from PI 342618B, and 2.66% were heterozygous. In contrast, for NJRINP, 46.07% were inherited from Nannong493-1, 50.06% were from PI 342618B, and the remaining 3.87% were heterozygous genotypes. The segregation ratios of each bin marker were calculated, and some significant segregation distortion regions were presented in both populations. In NJRINP, 176 of 5728 bins showed extreme segregation distortion at a significant level of *P* < 0.0001, distributed on Chr. 02, Chr. 16, and Chr. 19 (Supplementary Figure S4). In NJRI4P, one bin presented extreme segregation distortion at *P* < 0.0001, and two bins exhibited segregation distortion at *P* < 0.0005 (Supplementary Figure S4). Few markers showed segregation distortion among the two populations, indicating that the markers and the population were suitable for constructing a linkage map.

### Construction and Characterization of the Two Linkage Maps with Genomic Bin Markers

Two high-resolution linkage maps were constructed using genomic bin markers in JoinMap 3.0 software (van Ooijen and Voorrips, 2002). The linkage map of NJRINP was composed of 5,728 bin markers distributed on 20 chromosomes, spanning a total distance of 2,204.6 cM (**Table 3**; Supplementary Figure S5). The genetic distance between neighboring bins was approximately 0.4 cM, ranging from 0.0 to 7.5 cM, 95.1% of which were less than 0.5 cM, and there were only six regions greater than 3.0 cM between neighboring bins dispersed on Chr. 05 (bin1155-bin1156, bin1294-bin1341, bin1161-bin1077, bin1154-bin1171, and bin1171-bin1172) and Chr. 13 (bin3346-bin3458; **Figure 1D**; **Table 3**).

**Table 3:** Number of bin markers in each linkage group and the average distance.

Chr. (LG)	NJRINP				NJRI4P			
	No.	Distance	Range	Average	No.	Distance	Range	Average
Chr. 01 (D1a)	265	108.2	0.0–2.3	0.4	225	101.0	0.0–1.9	0.5
Chr. 02 (D1b)	304	124.9	0.0–2.0	0.4	258	127.4	0.0–6.4	0.5
Chr. 03 (N)	284	106.8	0.0–2.6	0.4	216	99.9	0.0–1.4	0.5
Chr. 04 (C1)	223	97.5	0.0–1.7	0.4	225	105.8	0.0–2.5	0.5
Chr. 05 (A1)	265	129.6	0.0–7.5	0.5	195	101.1	0.0–3.9	0.5
Chr. 06 (C2)	286	123.3	0.1–2.2	0.4	238	123.7	0.0–2.9	0.5
Chr. 07 (M)	270	104.3	0.1–1.9	0.4	226	105.0	0.0–2.3	0.5
Chr. 08 (A2)	353	125.4	0.1–2.6	0.4	294	135.5	0.2–2.3	0.5
Chr. 09 (K)	288	103.1	0.1–2.0	0.4	214	101.0	0.2–1.6	0.5
Chr. 10 (O)	304	110.6	0.0–1.8	0.4	237	125.4	0.0–2.3	0.5
Chr. 11 (B1)	254	98.6	0.0–2.2	0.4	191	88.3	0.0–2.0	0.5
Chr. 12 (H)	249	93.7	0.0–1.7	0.4	208	97.4	0.0–2.2	0.5
Chr. 13 (F)	365	133.6	0.1–7.0	0.4	259	144.8	0.2–14.1	0.6
Chr. 14 (B2)	265	93.8	0.1–2.1	0.4	194	88.0	0.2–2.0	0.5
Chr. 15 (E)	310	106.7	0.0–1.5	0.3	209	104.7	0.0–2.2	0.5
Chr. 16 (J)	237	87.4	0.0–1.7	0.4	172	85.6	0.0–2.7	0.5
Chr. 17 (D2)	308	116.1	0.0–1.9	0.4	220	103.1	0.2–2.1	0.5
Chr. 18 (G)	318	110.6	0.0–1.4	0.3	212	100.8	0.2–2.5	0.5
Chr. 19 (L)	305	120.4	0.1–1.9	0.4	201	99.4	0.2–2.3	0.5
Chr. 20 (I)	275	110.0	0.0–1.9	0.4	160	98.8	0.0–3.2	0.6
**Total**	5728	2204.6	0.0–7.5	0.4	4354	2136.7	0.0–14.1	0.5

*Chr., chromosome; LG, linkage group; No., number of bins; Distance, the genetic distance of LG in cM; Average, the average genetic distance between neighboring bins (cM).*

Another linkage map of NJRI4P was composed of 4,354 bins, covering 2,136.7 cM (**Table 3**; Supplementary Figure S6). There was no obvious difference in length between the two linkage maps. However, the average genetic distance between neighboring bin markers in NJRI4P was greater than that of NJRINP, with a value of 0.5 cM, and only 59.0% of interval regions were less than 0.5 cM (**Figure 1D**; **Table 3**) due to the small population size (161 lines) used in the construction of the linkage map. There were seven regions greater than 3.0 cM between two neighboring bins dispersed on Chr. 02 (bin228-bin229, bin322-bin323), Chr. 05 (bin1119-bin1118, bin986-bin925), Chr. 13 (bin2809-bin2808, bin2728-bin2810), and Chr. 20 (bin4263-bin4264; Supplementary Figure S6).

To compare the order of the bin markers, a dot-plot diagram (Supplementary Figures S7 and S8) was generated using the physical position of each marker on the Williams 82 genome against its genetic position on the linkage groups. According to the analyses of the two linkage maps, most of the markers showed good linear agreement between physical and genetic maps based on the basic framework. However, there were also five inverted regions, all of which were located at the end of the chromosomes, including the upper end of Chr. 05 and Chr. 13 and the lower end of Chr. 05, Chr. 11, and Chr. 14. The genome variations among different species might be the first reason for this non-uniformity.

The above results indicate that the features of the bin linkage maps were as follows: (i) the density of markers was high, totaling 5,728 and 4,354 bins in NJRINP and NJRI4P, respectively, with average genetic distance between neighboring bins of approximately 0.4 and 0.5 cM; (ii) evenness throughout the whole genome, with and average genetic distance between neighboring bins from 0.0–7.5 to 0.0–14.1 cM in the respective populations, 95.1 and 59.0% of which were less than 0.5 cM; and (iii) more bin markers in the inter-specific RIL populations. However, the bins relative to the RIL population were non-comparable among populations but comparable based on the reference genome.

### Mapping the Wild/Cultivated Gene/Alleles for Seed Bloom and Seed Coat Color

**FIGURE 2:**
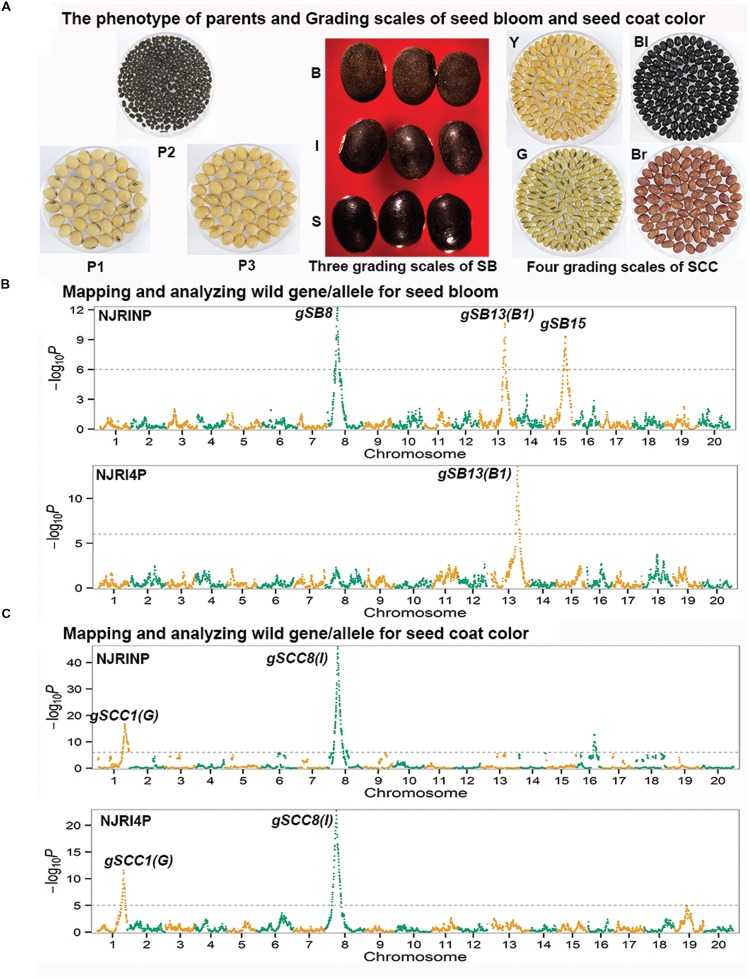
**Mapping and analysis of wild alleles for seed bloom (SB) and seed coat color (SCC) using NJRINP and NJRI4P.** The phenotypes of three parents and the grading scales of SB and SCC in NJRINP derived from Nannong86-4 (P1) and PI 342618B (P2) and NJRI4P derived from Nannong493-1 (P3) and PI 342618B (P2) are shown in **(A)**. The grading scales were as follows: S = shiny (absence of bloom), I = intermediate (between shiny and bloom), and B = bloom (heavy coating of powdery substance adhering to the seed coat) for SB, and Y = yellow, G = green, Bl = black, and Br = brown for SCC. The Manhattan plots on the left of the graph depict the extent of the association of 5,728 and 4,354 bin markers dispersed as shown over the 20 soybean chromosomes with SB **(B)** and SCC **(C)** of NJRINP and NJRI4P, respectively. The -log_10_*P* value is a measure of the degree to which a bin is associated with the trait based on the Chi-square test.

In the two RIL populations, two qualitative traits, SB and SCC, were classified into three and four grades (**Table 4**; **Figure 2A**), respectively. As shown in **Table 4**, for SB, 194, 40, and 42 lines were grouped into S (shiny, absence of bloom), B (bloom, heavy coating of powdery substance adhering to the seed coat) and I (intermediate, between shiny, and dull) in NJRINP, respectively, and the respective numbers were 79, 43, and 35 in NJRI4P. The associated loci of the two qualitative traits were mapped by the Chi-square test. As shown in **Figure 2B** and **Table 5**, a total of three genes were detected associated with SB in the two populations, among which *gSB13* was novelly mapped into a small region on Chr. 13, with the 1-LOD support interval of 33,911,568–34,322,119 bp in NJRINP and 33,689,175–34,469,255 bp in NJRI4P, corresponding to the locus of *B1* reported in a large genome region (Chen and Shoemaker, 1998). The other two genes of SB, *gSB8* and *gSB15*, were detected only in NJRINP, located in intervals of 8,144,404–8,643,358 bp on Chr. 08 and 22,763,999–42,462,013 bp on Chr. 15, respectively. The most significant bins in the 1-LOD interval of genes were used to analyze the effects of the wild/cultivated alleles. As shown in **Table 6**, all of the wild alleles (w) on the three loci were associated with the phenotype of I and B, except for *gSB15*, on which the wild allele occurred in two opposite directions at low frequency (0.10) in the I group and at high frequency (0.85) in the B group.

**Table 4:** Phenotypic distribution of three domestic traits among NJRINP and NJRI4P.

Trait	Environment	Parent		Range	Mean	*h*^2^ (%)
NJRINP		P1	P2			
SB	JP2010	*S*	*B*	*S* (192), *I* (42), *B* (40)		
SCC	JP2010	*Y*	*Bl*	*Y* (60), *G* (65), *Bl* (110), *Br* (1)		
DTF (d)	JP2010	50.3	40.0	48.3–63.3	50.8	92.4
	JP2011	38.0	32.0	32.5–49.0	37.2	94.5
	AH2012	51.3	31.7	32.3–56.3	42.6	92.5
	JP2013	49.7	32.3	34.0–60.0	44.3	92.6

**NJRI4P**		**P3**	**P2**			
SB	JP2010	*S*	*B*	*S* (79), *I* (35), *B* (43)		
SCC	JP2010	*Y*	*Bl*	*Y* (24), *G* (33), *Bl* (62), *Br* (20)		
DTF (d)	AH2012	52.3	31.3	36.7–60.0	44.7	89.3
	JP2012	50.7	35.0	40.0–54.0	48.1	94.8
	JP2013	51.3	32.3	34.0–56.7	41.3	95.1

*The two RIL populations. NJRINP and NJRI4P, were derived from Nannong86-4 (P1) and PI 342618B (P2) and from Nannong493-1 (P3) and PI 342618B (P2), respectively. Their performance, along with that of their parents in three evolutionary traits, seed bloom (SB), seed coat color (SCC) and days to flowering (DTF), are shown. The grading scales were as follows: *S* = shiny (absence of bloom), *I* = intermediate (between shiny and bloom), and *B* = bloom (heavy coating of powdery substance adhering to the seed coat) for SB, and *Y* = yellow, *G* = green, *Bl* = black, and *Br* = brown for SCC. The respective number of RILs is listed in parentheses. *h^*2*^*, heritability.*

**Table 5:** Genes/QTLs associated with three domestic traits in the two populations.

Gene/QTL	Pop	Env.	Chr. (LG)	Position	Interval (bp)	LOD	*R^2^*	Add	Published gene
**SB**									
***gSB8***	**NP**	**JP2010**	**8 (A2)**	**36.3**	**8144404–8643358**	**12.2**		***B***	
*gSB13*	NP	JP2010	13 (F)	90.2	33911568–34322119	10.6		*B*	*B1*
	4P	JP2010	13 (F)	103.3	33689175–34469255	5.3		*B*	
***gSB15***	**NP**	**JP2010**	**15 (E)**	**75.8**	**22763999–42462013**	**9.3**			
**SCC**									
*gSCC1*	NP	JP2010	1 (D1a)	91.6	52279678–52646512	16.8		*G*	***G***
	4P	JP2010	1 (D1a)	65.9	52032052–52462360	11.6		*G*	
*gSCC8*	NP	JP2010	8 (A2)	36.7	8346892–8643359	46.2		*Bl*	*I*
	4P	JP2010	8 (A2)	40.0	8434875– 8540484	22.7		*Bl*	
**DTF**									
***qDTF02***	**4P**	**AH2012**	**2 (D1b)**	**0.2**	**0–233022**	**5.8**	**10.0**	**1.8**	
		JP2012	2 (D1b)	0.2	0–233022	3.7	9.0	1.0	
		JP2013	2 (D1b)	0.2	0–233022	6.2	10.8	2.0	
*qDTF10*	NP	JP2010	10 (O)	83.5	44593532–45047245	25.2	26.4	1.6	*E2*
		JP2011		83.1	44593532–45010360	40.5	36.0	2.1	
		JP2013		83.1	44593532–44937701	37.8	28.6	3.2	
		AH2012		83.1	44593532–44937701	55.3	44.3	3.3	
	4P	AH2012		101.8	44673399–46260105	11.0	19.5	2.0	
		JP2012		99.9	44673399–45833532	6.4	14.2	1.2	
		JP2013		101.8	45105038–46425953	7.0	12.0	2.0	
***qDTF13***	**4P**	**AH2012**	**13 (F)**	**94.5**	**31943630–32681690**	**4.8**	**7.7**	**1.2**	
		JP2013		95.9	30886271–33458132	3.0	4.9	1.2	
*qDTF16.1*	4P	JP2012	16 (J)	0.6	0–1139451	3.9	8.6	-0.9	*Fflr13-8*
		JP2013		9.8	751606–3823243	3.5	5.7	-1.3	
*qDTF16.2*	NP	JP2010	16 (J)	22.3	3966960–4570856	9.3	8.3	-0.9	*GmFT5a; GmFT3a*
		JP2011		22.3	3966960–4409445	15.2	10.2	-1.1	
		JP2013		22.3	3966960–4409445	21.6	13.5	-2.2	
		AH2012		22.3	3929247–4347585	23.8	13.2	-1.8	
*qDTF19*	NP	JP2010	19 (L)	107.3	47310112–47815831	11.4	10.4	-1.0	*E3*
		JP2011		107.3	47394320–47757695	17.8	12.3	-0.6	
		JP2013		107.3	47554129–47757695	28.4	19.2	-2.6	
		AH2012		107.3	47442735–47757695	20.2	11.1	-1.7	
	4P	AH2012		89.3	47083725–47636059	3.2	5.0	-1.0	
		JP2013		89.3	46456849–48190855	4.5	7.4	-1.5	

*SB, SCC, and DTF represent the three traits (SB, SCC, and DTF, respectively), and the QTLs in boldface represent the novel QTLs detected in wild soybean in the present paper. In the Pop column, NP and 4P represent the populations derived from Nannong86-4 × PI 342618B and Nannong493-1 × PI 342618B, respectively. Env., environment, in which JP2010, JP2011, JP2013, AH2012, and JP2012 represent the five environments that were used to evaluate the phenotype of the mapping populations. Position, the genetic position (cM) of the genes/QTLs on the bin maps of NJRINP and NJRI4P. Chr. (LG), chromosome (linkage group); Interval, one-lod-support interval of gene/QTL. *R^*2*^*, percentage of the phenotypic variation explained by an individual QTL. Add: additive effect of allele derived from the wild soybean; B, SB; G and Bl, green and black SCC, respectively. In the Published column, the genes/QTLs reported in a previous study are listed.*

**Table 6:** Analysis the effect of wild allele of the genes conferring SB and SCC.

Trait/population	Gene/allele	Grading scales (Percentage)			
SB		*S*	*I*	*B*	
NJRINP	*gSB8*				
	*w*	54 (0.28)	35 (0.85)	28 (0.7)	
	*c*	140 (0.72)	6 (0.15)	12 (0.3)	
	*gSB13*				
	*w*	79 (0.41)	32 (0.78)	38 (0.95)	
	*c*	116 (0.60)	9 (0.22)	1 (0.03)	
	*gSB15*				
	*w*	90 (0.46)	4 (0.10)	34 (0.85)	
	*c*	104 (0.54)	38 (0.90)	6 (0.15)	
	**Total**	194 (1.00)	42 (1.00)	40 (1.00)	

NJRI4P	*gSB13*				
	*w*	19 (0.24)	34 (0.97)	35 (0.81)	
	*c*	60 (0.76)	1 (0.03)	8 (0.19)	
	**Total**	79 (1.00)	35 (1.00)	43 (1.00)	

**SCC**		***Y***	***G***	***Bl***	***Br***
NJRINP	*G*, *I*				
	*w, w*		2 (0.03)	50 (0.45)	
	*w, c*	3 (0.05)	56 (0.86)		
	*c, w*			58 (0.53)	1 (1.00)
	*c, c*	57 (0.95)	7 (0.11)	2 (0.02)	
	**Total**	60 (1.00)	65 (1.00)	110 (1.00)	1 (1.00)

NJRI4P	*G*, *I*				
	*w, w*		1 (0.03)	27 (0.44)	3 (0.15)
	*w, c*		30 (0.91)	1 (0.02)	5 (0.25)
	*c, w*			34 (0.55)	4 (0.20)
	*c, c*	24 (1.00)	2 (0.06)		8 (0.40)
	**Total**	24 (1.00)	33 (1.00)	62 (1.00)	20 (1.00)

*NJRINP and NJRI4P represent the RIL populations derived from Nannong86-4 × PI 342618B and Nannong493-1 × PI 342618B, respectively. SB and SCC represent SB and seed coat color (SCC), respectively, and their grading scales were as follows: *S* = shiny (absence of bloom), *I* = intermediate (between shiny and bloom), and *B* = bloom (heavy coating of powdery substance adhering to the seed coat) for SB, and *Y* = yellow, *G* = green, *Bl* = black and *Br* = brown for SCC. In each sub-table, *w* and *c* represent the wild and cultivated alleles at each locus, respectively; the number outside of the parentheses is the number of RILs in each genotypic group, and the number in parentheses is the percentage of total lines in each phenotypic group. The interaction of two seed coat genes, *G* and *I*, can be illuminated through the allele effect analysis table at the molecular level.*

With the same method as used for SB, a total of three genes were detected for SCC in the two populations. Among them, *gSCC1* and *gSCC8* corresponding to the two SCC genes, *G* (Song et al., 2004) and *I* (Ohnishi et al., 2011), respectively, were mapped into two small regions of 52,279,678–52,646,512 and 8,346,892–8,643,359 bp in NJRINP and 52,032,052–52,462,360 and 8,434,875–8,540,484 bp in NJRI4P on Chr. 01 and Chr. 08, respectively (**Figure 2C**; **Table 5**). The effects of wild alleles on the two loci are shown in **Table 6**. In NJRINP, a high frequency of the wild allele (0.86) of *G* was observed in 65 RILs with green SCC, and the wild allele of *I* was associated with black SCC with a frequency of 0.98 in 116 black SCC RILs. The fact that the 50 RILs of NJRINP with the genotype w/w at the loci of *G* and *I* were associated with the black SCC instead of the green SCC indicates that there must be interaction between these loci, and this interaction was verified in NJRI4P. *G* and *I* can explain 93.6% of the phenotypic variation in NJRINP, including 57 yellow, 56 green, 50 black, and 58 black SCC lines of a total of 221 of 236 lines; the third gene, *gSCC16*, detected on Chr. 16 in NJRINP, whose wild allele is associated with both green and black SCC, might be a false-positive gene for SCC. In summary, two genes, *G* and *I*, were detected in the two mapping populations, *G* was mapped to a smaller region than that previously reported, and the interaction between these genes was verified first at the molecular level.

### Mapping the Wild/Cultivated QTL Alleles Conferring Days to Flowering

#### Phenotypic and Genotypic Variation of Days to Flowering

Days to flowering was evaluated for NJRINP and NJRI4P populations under four and three environments, respectively. As shown in **Table 4**, large differences and broad phenotypic variations were observed between the cultivated and wild parents and among their inter-specific RIL populations in DTF (Supplementary Figure S9). For NJRINP, the ranges of DTF values were 48.3–63.3, 32.5–49.0, 32.3–56.3, and 34.0–60.0 days in the environments of JP2010, JP2011, AH2012, and JP2013, respectively, with means of 50.8, 37.2, 42.6, and 44.3 days. For NJRI4P, the ranges of DTF were 36.7–60.0, 40.0–54.0, and 34.0–56.7 days in AH2012, JP2012, and JP2013, respectively, with means of 44.7, 48.1, and 41.3 days. The high heritability of DTF from 89.3 to 95.1% suggests that genetic variation accounted for a major part of the phenotypic variance in the populations. A joint ANOVA of the data from multiple environments showed that the variations among RILs, environments, and RIL × environment interactions were all significant (Supplementary Table S3). Therefore, QTL mapping was performed separately based on individual environments.

#### QTL Mapping of Days to Flowering

**FIGURE 3:**
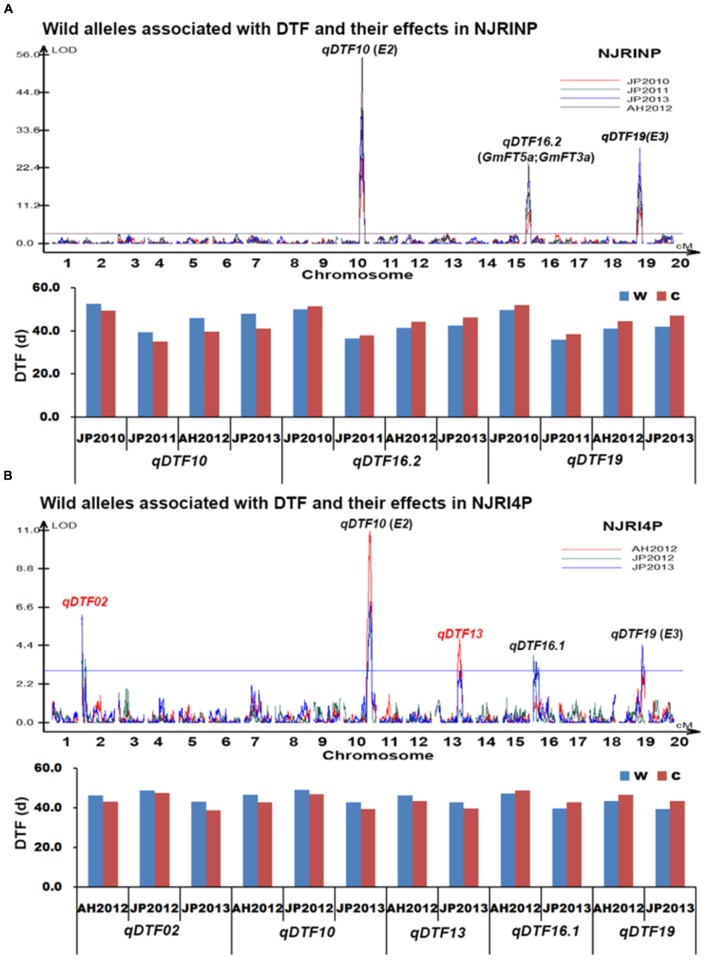
**Wild alleles and their effects associated with days to flowering (DTF) in NJRINP and NJRI4P. (A)** Wild alleles and their effects associated with DTF in NJRINP derived from Nannong86-4 and PI 342618B. **(B)** Wild alleles and their effects associated with DTF in NJRI4P derived from Nannong493-1 and PI 342618B. A total of six wild alleles/QTLs related to DTF were detected in the two populations, among which *qDTF10* and *qDTF19* could be detected in both of the populations under all environments, including JP2010, JP2011, JP2012, AH2012 and JP2013, and encompass the cloned genes *E2* and *E3*, respectively. The locus *qDTF16.2*, detected only in NJRINP under four environments, also encompassed the two linked *FLOWERING LOCUS T* genes *GmFT5a* and *GmFT3a.* The other three QTLs, including two newly identified QTL/alleles, *qDTF02* and *qDTF13*, and one previously reported locus, *qDTF16.1*, were detected only in NJRI4P. The allele effects were analyzed according to the genotype of lines based on the most significant bin marker, and two letters, *w* and *c*, represent the wild and cultivated alleles, respectively.

With the composite interval mapping using WinQTLCart 2.5 software (Wang et al., 2003), a total of three putative QTLs were identified in the NJRINP (**Table 5**; **Figure 3A**). The QTL *qDTF10* in the region of 44,593,532–45,047,245 bp on Chr. 10, overlapping with the previously cloned gene *E2*, had the highest genetic contribution values (*R*^2^) ranging from 26.4 to 44.3% in the four different environments. *qDTF16.2* in the region of 3,966,960–4,570,856 bp on Chr. 16 and *qDTF19* in the region of 47,310,112–47,815,831 bp on Chr. 19 were stable across the four environments, with *R*^2^ values ranging from 8.3–13.5 to 11.1–19.2%, respectively, and overlapped with two linked *FLOWERING LOCUS T* homologs of *Arabidopsis thaliana* (*GmFT5a* and *GmFT3a*; Kong et al., 2010) and *E3*, respectively. The wild alleles on these loci were associated with additive effects from -2.6 to 3.3 days. To verify the allelic differences on each locus, the wild allele effects of DTF were also roughly estimated from the individuals with and without the allele according to the most significant bin marker genotypes. As shown in **Figure 3A**, there were significant differences between wild and cultivated alleles at each locus, with the *G. soja* allele (w) causing a consistently later DTF than that of the *G. max* allele over time on *qDTF10* and earlier DTF on *qDTF16.2* and *qDTF19*.

In the NJRI4P population, a total of five QTLs were detected in the three environments (**Table 5**; **Figure 3B**), located on Chr. 02, 10, 13, 16 and 19, with *R*^2^ values ranging from 4.9 to 19.5%. All of the QTLs could be detected in at least two environments. Among these QTLs, *qDTF10* and *qDTF19* corresponded to the reported genes *E2* and *E3*, respectively, and *qDTF16.2* spanned two physical intervals of 1–1,139,451 and 751,606–3,823,243 bp in the environments of JP2012 and JP2013 and overlapped with the previously reported QTL *Fflr 13-8* (Pooprompan et al., 2006). However, these QTLs were narrowed down to intervals of 1.1 and 3.1 Mb, respectively, in the NJRI4P population. The other two QTLs, *qDTF02* and *qDTF13*, were newly detected in the wild soybean PI 342618B and mapped to intervals of 1–233,022 and 30,886,271–33,458,132 bp on Chr. 02 and Chr. 13, respectively. The wild alleles at the five loci were associated with additive effects from -1.5 to 2.0 days based on the mapping software, consistent with the allele effects of DTF that were roughly estimated from the individuals with or without wild alleles (**Figure 3B**). Compared to the cultivated alleles, the *G. soja* alleles (w) caused a consistently later DTF on *qDTF02*, *qDTF10*, and *qDTF13* and an earlier DTF on *qDTF16.1* and *qDTF19*.

In conclusion, a total of six QTLs were detected for DTF. In previous research, three QTLs, *qDTF10*, *qDTF16.1* and *qDTF19*, could be detected in other mapping populations derived from *G. max* soybeans, such as ‘Misuzudaizu × Moshidou Gong 503’ for *qDTF10* and *qDTF19* (Yamanaka et al., 2001) and ‘AGS292 × K3’ for *qDTF16.1* (Pooprompan et al., 2006), indicating that allele differentiations existed not only between *G. max* and *G. soja* at these loci for DTF but also among *G. max* soybeans. The QTL *qDTF16.2* overlapped with two linked *FLOWERING LOCUS T* homologs of *A. thaliana* (*GmFT5a* and *GmFT3a*; Kong et al., 2010). The other two QTL, *qDTF02* and *qDTF16.1*, were newly identified in the wild soybean, which indicated that allele differentiations were not detected in cultivated soybeans at these loci for DTF and that these wild alleles might have the potential to broaden the genetic basis of cultivated soybean in DTF.

### Candidate Gene Prediction for the Three Evolutionary Traits Using the High-Density Bin Maps

Seed coat pigmentation is induced by the deposition of a number of flavonoids in the seed coat of soybean, and the synthesis of these compounds mainly derives from an anthocyanin biosynthesis branch of the phenylpropanoid pathway of secondary metabolism (Yang et al., 2010). According to the mapping results in the present study, there were a total of 54 gene models in the interval of *gSCC1* (*G*) (**Table 5**), among which only one gene model of *Glyma01g40590* was predicted to involve in anthocyanin accumulation and thus might be the most likely candidate gene of *gSCC1* (*G*). According to the gene annotation database at SoyBase^1^, the small physical interval of *gSCC8* in the NJRINP population encompassed only 63 gene models, among which *Glyma08g11520*, *Glyma08g11530*, *Glyma08g11610*, *Glyma08g11620*, *Glyma08g11630*, and *Glyma08g11650* were predicted to encode *Chalcone* and *Stilbene synthase* family proteins and thus might be the candidate genes of *gSCC8* (*I*) (**Table 5**).

The key genes and distinct pathways involved in flowering have been identified through many *Arabidopsis* studies. *Phytochromes* are red-light- and far-red-light-absorbing photoreceptors in the leaves of most plants (Chen et al., 2004). Mutations in the *phytochrome* genes cause altered flowering phenotypes in *Arabidopsis* and soybean (Reed et al., 1994; Liu et al., 2008). In the region of the novel locus *qDTF16.1* detected in the wild soybean, there was only one soybean gene (*Glyma16g03030*) orthologous to *Arabidopsis SPA1* (suppressor of *phyA* protein family). *APETALA 2* (*AP2*) is related to various aspects of plant development, including flowering and seed mass control (Jofuku et al., 2005). In the interval of *qDTF13*, three gene models, *Glyma13g28810*, *Glyma13g29920* and *Glyma13g30710*, were predicted to encode the *AP2* domain. A total of 19 gene models were located in the interval of *qDTF02*, and although there were no homologs of genes for *Arabidopsis* flowering, one gene model, *Glyma02g00300*, was predicted to be involved in the response to red or far red light. These candidate genes are worth investigating in future research.

## Discussion

### The Features of a RAD-seq Based Genome-Wide Inter-specific Bin Linkage Map

The marker density is high in the two inter-specific linkage maps. In the past two decades, at least 40 genetic maps have been constructed^1^ that provide good tools for investigating the soybean genome. However, many of them were constructed based on morphological, cytological, biochemical and SSR markers, among others, which are limited in number or are difficult to genotype if 100 or 1000s of markers are involved in a large population. Because there have been few markers in most previous research, the QTLs of target traits were mapped to large intervals of more than 10 cM. Thus, the bottlenecks of soybean map-based cloning or fine mapping included the insufficiency of molecular markers and the lack of highly efficient genotyping approaches, as suggested by Xu et al. (2013). Recent advances in next-generation sequencing technologies have provided effective platforms for the direct detection of high-density SNP markers, which are the most abundant DNA variations, to genotype a mapping population (Varshney et al., 2009; Chen et al., 2014; Li et al., 2015). Using whole genome re-sequencing technology, a linkage map composed of 3,509 bin markers had been constructed using two *G. max* accessions (Xu et al., 2013). Because of the expense of the whole-genome re-sequencing of a large population, a RAD-seq technology was employed in the present study. According to the breakpoint information of each line, the highest-density linkage maps were constructed, including 5,728 and 4,354 bin markers in NJRINP and NJRI4P, respectively, which were denser than those in previous studies. The above results suggest that the RAD-seq-based bin strategy was efficient for the generation of a high-density linkage map.

There were many polymorphisms at the phenotype and genome levels among the two populations. Although, there are many advantages using an inter-specific cross to a construct linkage map (Miller and Tanksley, 1990), the number of inter-specific maps is much lower because of the laborious process of their development. In the present research, two ultra-high-density genetic maps were constructed using two cultivated soybeans and a shared wild soybean. According to the results, there were a wide range of polymorphisms between the two inter-specific parents, including DNA and phenotype. At the DNA level, 828,796 and 736,076 SNPs were polymorphic between Nannong86-4 and PI 342618B and between Nannong493-1 and PI 342618B, respectively, much more than that (251,612) between Nannong86-4 and Nannong493-1. This indicates that nucleotide diversity decreased remarkably during the domestication process from wild to cultivated lines. This might be the reason why much more bins were contained in the present populations than in the population derived from the two cultivars Magellan and PI 438489B (Xu et al., 2013), although whole-genome sequencing was used to identify SNPs in the population. The polymorphisms between the parents in phenotype determine the application scope of the mapping population. In the present study, there were large differences between Nannong86-4, Nannong493-1 and wild soybean PI 342618B, including the color of the flower and seed coat, stem growth habit, DTF, seed size, protein and oil contents, stress tolerance and so on. Thus, the two populations would have wide ranges of applications.

The two linkage maps had high quality and mapping accuracy. Compared to the reference genome Wm82, the orders of most bins in each linkage map of NJRINP and NJRI4P showed good linear agreement with their physical positions. Using the two inter-specific maps, seven well-studied loci, *B1* for SB (Chen and Shoemaker, 1998), *G* (Song et al., 2004) and *I* (Ohnishi et al., 2011) for SCC, and *E2* (Watanabe et al., 2011), *E3* (Watanabe et al., 2009), *qDTF16.1* (Pooprompan et al., 2006) and two linked *FLOWERING LOCUS T* (Kong et al., 2010) for DTF, were detected, indicating the high quality of the two maps. The mapping accuracy was effectively improved, and few genes were contained in the 1-LOD interval of genes/QTLs. If the most significant bin marker that is associated with trait is used to predict candidate genes, the number of genes will be much lower. Thus, in the present paper, the candidate genes of traits can be predicted for subsequent deep research, such as gene cloning and gene functional verification, e.g., *G* was narrowed to a region of 0.4 Mb on Chr. 01, encompassing 54 gene models, among which only *Glyma01g40590* was predicted to be involved in anthocyanin accumulation. Due to the high resolution of gene/QTL mapping, interactions between genes/QTLs can be detected. For example, the interaction of two SCC genes, *G* and *I*, were first detected and analyzed at the molecular level in the present study and verified by both of the populations.

### The Regions of Differentiation between Wild and Cultivated Soybean with Regard to Two Qualitative Traits and One Quantitative Trait

The classic characteristics of the wild soybean seed coat are bloom and black color. Through evolution and artificial selection, the SB disappeared and SCC became yellow, but the location of these wild alleles is largely unknown. Although, three genes of SB, *B1*, *B2* and *B3*, have been reported (Palmer et al., 2004), only *B1* has been mapped to a large region of Chr. 13 (Chen and Shoemaker, 1998), while the position of other two loci are unknown. In the present study, three genome regions had allelic differentiation between wild and cultivated soybean in SB, among which *gSB13* was located in the region of *B1*, while the other two genes may be in the region of *B2* or *B3*. So far, at least five genetic loci (*I*, *T*, *W1*, *R*, and *O*) are known to control SCC (Palmer et al., 2004), but only two loci that correspond to *G* and *I* were detected to have allelic differentiation between the wild and cultivated parents in the present study. The two loci explained almost the entire genetic variation of RILs with effects of green and black SCC, but the gene associated with brown SCC was not detected in the two RIL populations, which might be due to the low frequency of brown SCC. Meanwhile, we first detected the interaction between *G* and *I* in both populations at the molecular level.

Worldwide, soybean is grown at a wide range of latitudes from the equator to up to 50°, most likely due to genetic diversity at a large number of genes and QTLs controlling DTF. Several DTF genes, designated as *E1*–*E8* (Bernard, 1971; Buzzell, 1971; Buzzell and Voldeng, 1980; Mcblain and Bernard, 1987; Bonato and Vello, 1999; Cober and Voldeng, 2001; Cober et al., 2010) and *J* (Ray et al., 1995), have been characterized by classical methods in cultivated soybeans. Of these, *E1*, *E2*, *E3*, and *E4* have been cloned (Liu et al., 2008; Watanabe et al., 2009; Watanabe et al., 2011; Xia et al., 2012). Moreover, numerous QTLs for DTF were detected by linkage mapping and genome-wide association studies, of which 63 QTLs (Mansur et al., 1996; Orf et al., 1999; Yamanaka et al., 2000; Tasma et al., 2001; Funatsuki et al., 2005; Reinprecht et al., 2006) are published in SoyBase^2^. Despite the economic importance of soybean, our knowledge about the molecular mechanisms of its flowering is still limited, especially in wild soybean. Here, we show the identification of DTF loci in a wild soybean and present a total of six QTLs that were identified in the two populations in multiple environments. Among them, *qDTF10*, *qDTF16.2*, and *qDTF19* overlapped the cloned DTF genes *E2* (Watanabe et al., 2011), *GmFT5a* and *GmFT3a* (Kong et al., 2010), *E3* (Watanabe et al., 2009), respectively, in cultivated soybean, and *qDTF16.1* was detected by Pooprompan et al. (2006) in a *G. max* cross, suggesting that allelic differentiation occurs not only in cultivated soybeans but also between *G. max* and *G. soja* in the four regions of DTF genes. The other two genes were newly detected in wild soybean; allelic differentiation in cultivated soybeans has been not detected but might occur only between *G. max* and *G. soja*, which may have the potential to broaden the genetic basis of DTF in cultivated soybean.

## Author Contributions

WW and ML carried out the phenotyping and genotyping of the two RIL populations, participated in the construction of the populations and data analysis, and drafted the manuscript. YW participated in the sequencing library construction, SNP calling and bin-map construction. XL, SC, and LS participated in the phenotyping of the two populations, and JK participated in language editing. TZ and JG conceived the study, participated in its design and coordination, and helped to draft the manuscript. All of the authors read and approved the final manuscript.

## Conflict of Interest Statement

The authors declare that the research was conducted in the absence of any commercial or financial relationships that could be construed as a potential conflict of interest.

## Footnotes

1 http://www.soybase.org/

2 http://www.soybase.org/
