# Supplementary material for: Characterizing Two Inter-specific Bin Maps for the Exploration of the QTLs/Genes that Confer Three Soybean Evolutionary Traits
Source: Front Plant Sci. 2016 Aug 23;7:1248. doi: 10.3389/fpls.2016.01248 (PMC4994327; doi:10.3389/fpls.2016.01248)
Supplement: Supplementary file 1 [file Data_Sheet_1.DOC]

Supplementary Material

# Characterizing two inter-specific bin maps for the exploration of the QTL/genes that confer three soybean evolutionary traits

Wubin Wang^1†^, Meifeng Liu^1†^, Yufeng Wang^1^, Xuliang Li^1^, Shixuan Cheng^1^, Liping Shu^2^, Zheping Yu^1^, Jiejie Kong^1^, Tuanjie Zhao^1^*, Junyi Gai^1^*

*** Correspondence:** Tuanjie Zhao: [tjzhao@njau.edu.cn](mailto:tjzhao@njau.edu.cn); Junyi Gai: [sri@njau.edu.cn](mailto:sri@njau.edu.cn)

# Supplementary Figures and Tables

## Supplementary Figures


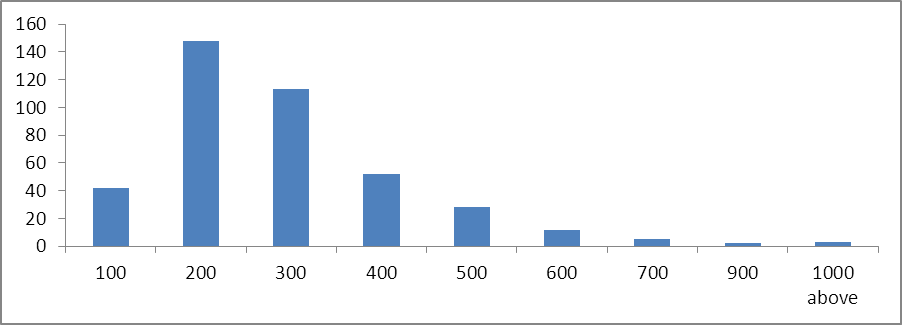


**Figure S1. The raw sequencing data of 455 lines.** The X axis indicates the range of Megabase (Mb); the Y axis indicates the number of lines fall in the range of data.


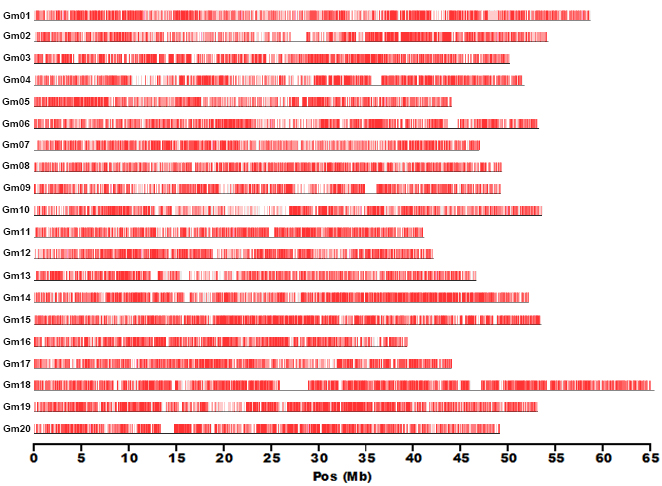


**Figure S2. The distribution of SNPs identified in NJIRNP population.** Pos**,** the physical position of bins on the referenced genome of Wm 82.


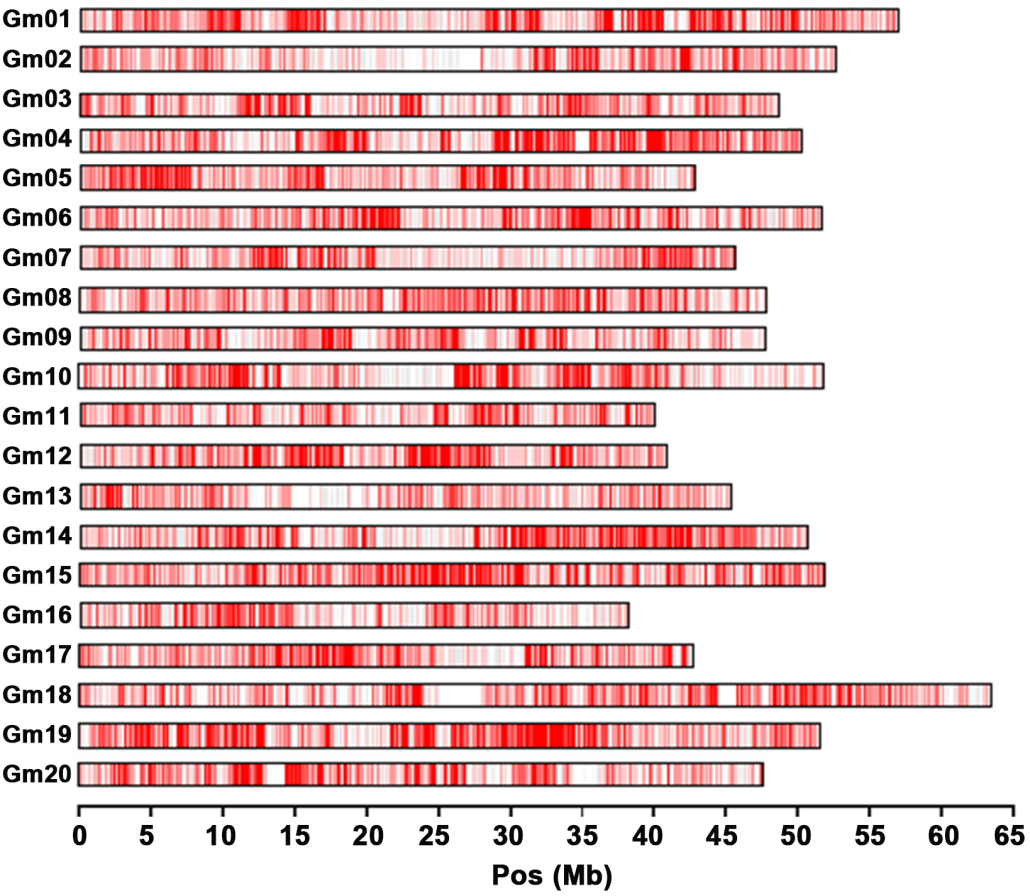


**Figure S3. The distribution of SNPs identified in NJIR4P population.** Pos**,** the physical position of bin on the referenced genome of Wm 82.


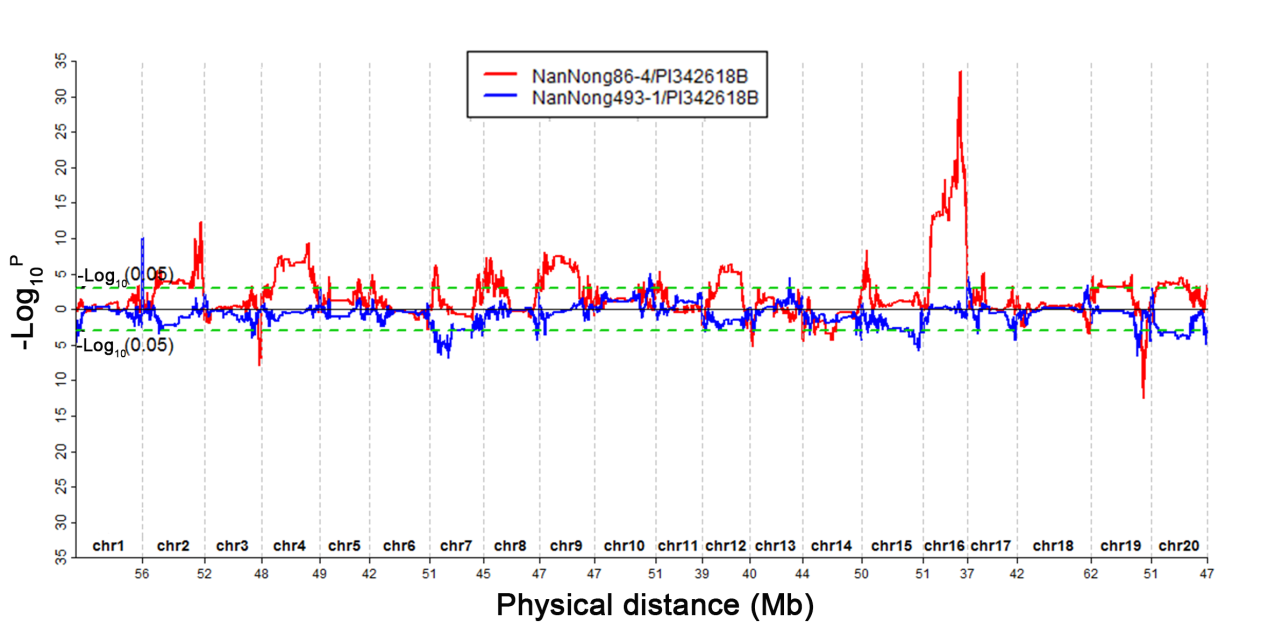


**Figure S4. Negative log_10_*^P^* values of segregation ratios in the two populations**

**Figure S5. The genetic map of NJRINP derived from Nannong 86-4 and PI 342618B.**

Figure S5 continued

**
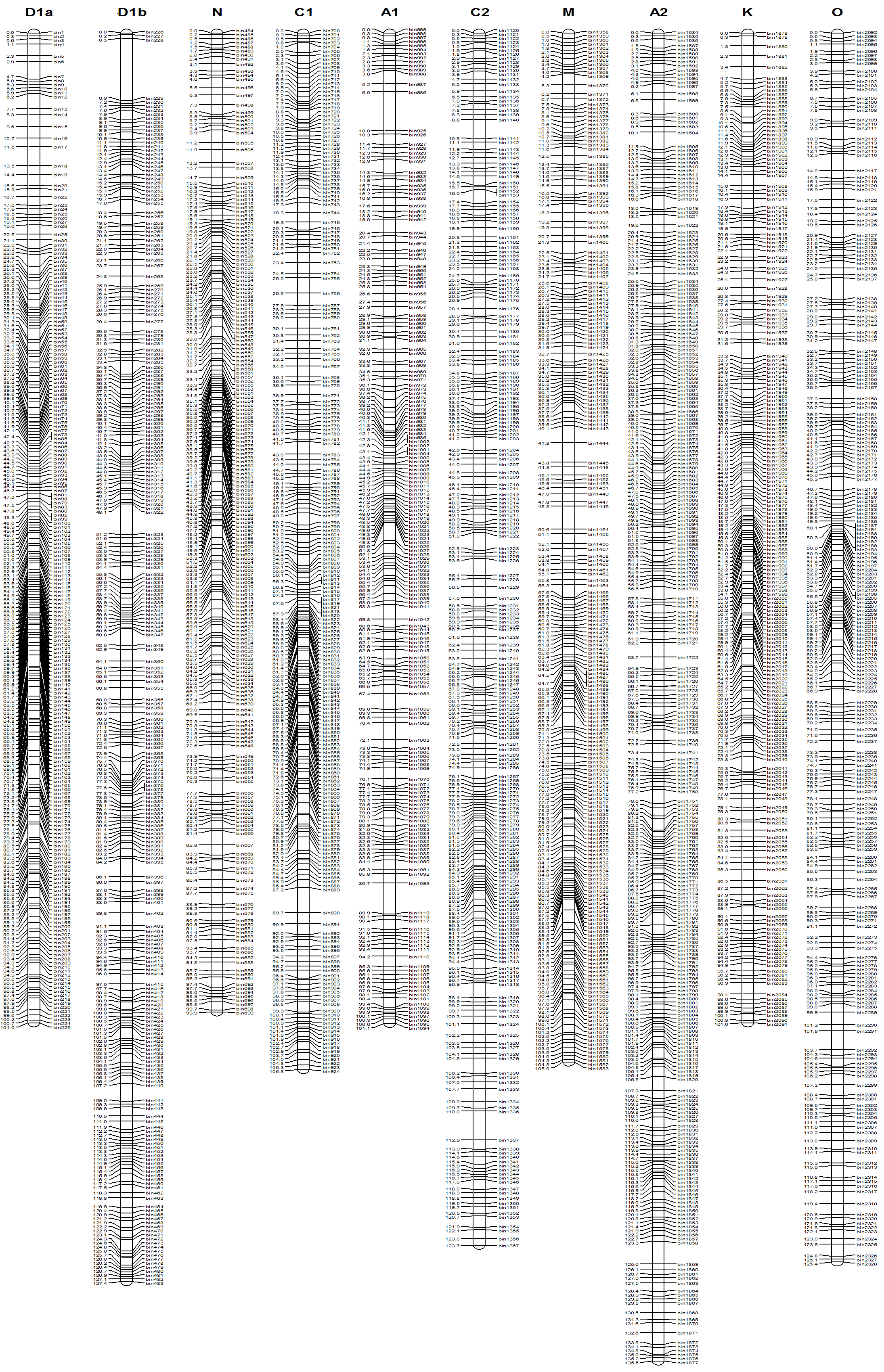
 Figure S6. The genetic map of NJRI4P derived from Nannong 493-1 and PI 342618B**


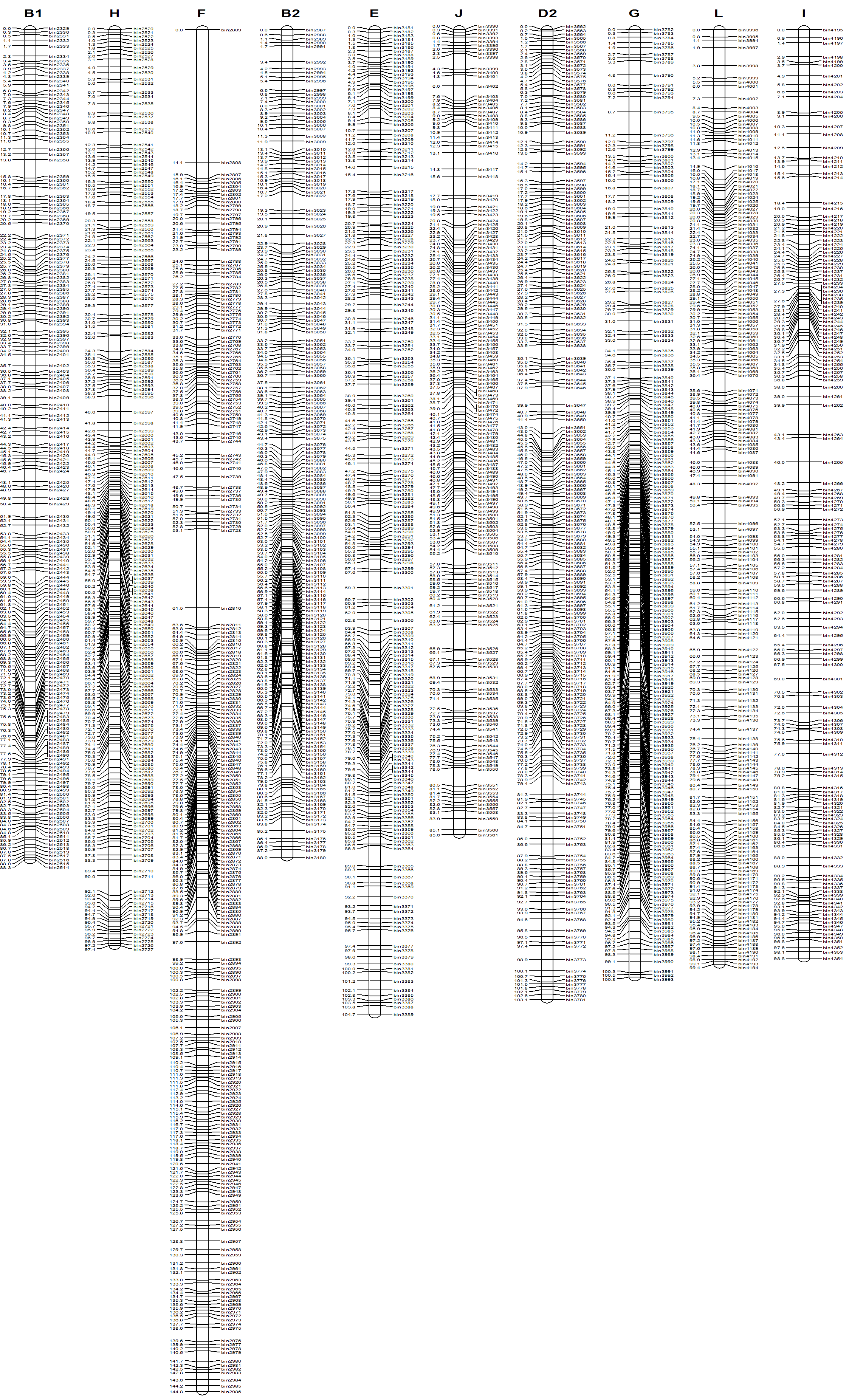


**Figure S6**. Continued


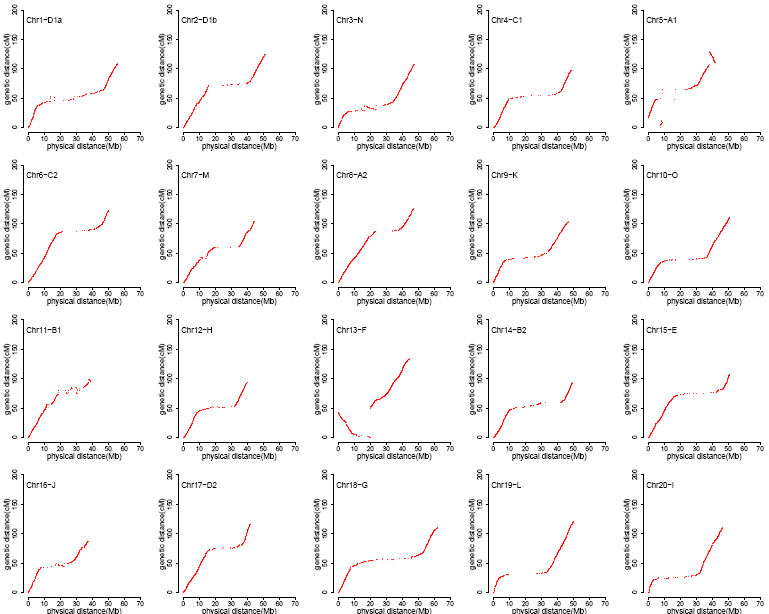


**Figure S7. Collinear analysis of the consensus between genetic and physical maps in NJRINP.** The X axis indicates the physical position of each bin marker; the Y axis indicates the genetic position of each bin marker.


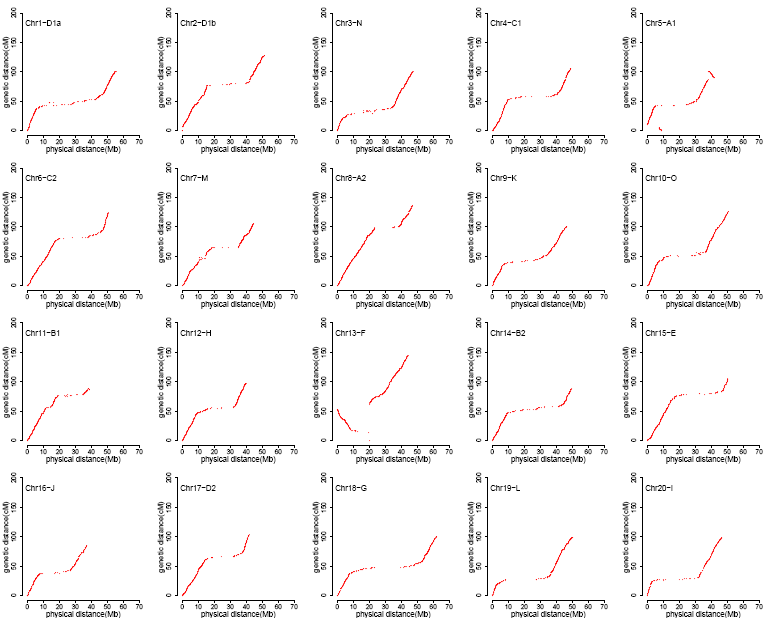


**Figure S8. Collinear analysis of the consensus between genetic and physical maps of NJRI4P.** The X axis indicates the physical position of each bin marker; the Y axis indicates the genetic position of each bin marker.


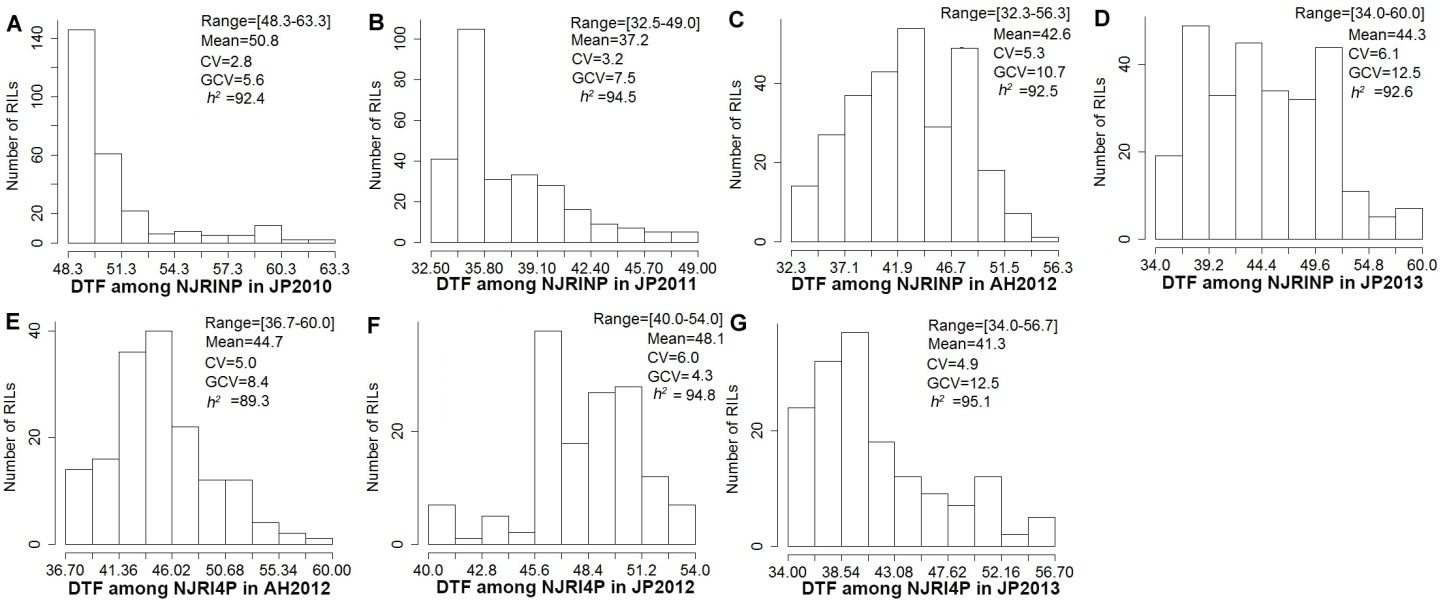


Figure S9. Frequency distributions of days to flowering in NJRINP and NJRI4P. *CV*: coefficient of variation; *GCV*: genetic coefficient of variation; *h^2^*, heritability. A, B, C, D represents the distribution of days to flowering (DTF) among NJRINP in the environments of JP2010, JP 2011, AH2012 and JP2013, respectively. E, F, G represents the distribution of DTF among NJRI4P in the environments of AH2012, JP2012 and JP2013, respectively.

## Supplementary Tables

Table S1. The whole genome re-sequencing information of the three parents

| Parent | Total reads (M) | Total bases (Gb) | Mapped reads (M) | Mapped bases (Gb) | Coverage (%) | Average depth (X) |
| --- | --- | --- | --- | --- | --- | --- |
| PI342618B | 123.52 | 11.12 | 111.23 | 10.01 | 86.48 | 9.89 |
| Nannong 86-4 | 134.42 | 12.10 | 125.24 | 11.27 | 93.82 | 10.69 |
| Nannong 493-1 | 110.36 | 9.93 | 101.49 | 9.13 | 87.57 | 9.13 |

Table S2. Distributions of physical lengths of bins and genetic distances between neighboring bins in NJRINP and NJRI4P

| Population | Physical bin length | | | | | | | | | | | Mean | Range |
| --- | --- | --- | --- | --- | --- | --- | --- | --- | --- | --- | --- | --- | --- |
|  | Midpoint (kbp) | | | | | | | | | | |  |  |
|  | 100 | 200 | 300 | 400 | 500 | 600 | 700 | 800 | 900 | 1000 | >1050 |  |  |
| NJRINP | 4632 | 585 | 181 | 72 | 42 | 30 | 22 | 22 | 16 | 10 | 116 | 165.8 | 1.5-17704.5 |
| NJRI4P | 3082 | 678 | 226 | 91 | 42 | 34 | 25 | 15 | 17 | 19 | 125 | 218.2 | 30.0-18886.5 |
|  | Genetic distance between neighboring bins | | | | | | | | | | | Mean | Range |
|  | Midpoint (kbp) | | | | | | | | | | |  |  |
|  | 0.25 | 0.75 | 1.25 | 1.75 | 2.25 | 2.75 | >3 |  |  |  |  |  |  |
| NJRINP | 4477 | 982 | 174 | 55 | 12 | 3 | 6 |  |  |  |  | 0.4 | 0.0-7.5 |
| NJRI4P | 2558 | 1418 | 280 | 51 | 15 | 5 | 7 |  |  |  |  | 0.5 | 0.0-14.1 |

Table S3. Analysis of variance of days to flowering in NJRINP and NJRI4P

| Source | NJRINP | | |  | NJRI4P | |  |
| --- | --- | --- | --- | --- | --- | --- | --- |
|  | DF | MS | F |  | DF | MS | F |
| Environment | 3 | 20347 | 209.88** |  | 2 | 3699.53 | 111.70** |
| Rep (Environment) | 7 | 88.23 | 20.69** |  | 6 | 29.92 | 5.63** |
| RIL | 283 | 160.41 | 12.29** |  | 160 | 108.59 | 10.40** |
| RIL × Year | 820 | 13.33 | 3.13** |  | 299 | 10.65 | 2.00** |
| Error | 1766 | 4.26 |  |  | 662 | 5.32 |  |
| Total | 2879 |  |  |  | 1129 |  |  |

F-tests were carried out by using GLM procedure under Model III, where RIL and Year were treated as random effects; ** indicates significant at 1% level; Rep (Environment) means replications within Environment.
